# Design and Preparation of a Biobased Colorimetric pH Indicator from Cellulose and Pigments of Bacterial Origin, for Potential Application as Smart Food Packaging

**DOI:** 10.3390/polym14183869

**Published:** 2022-09-15

**Authors:** Lúcia F. A. Amorim, Ana P. Gomes, Isabel C. Gouveia

**Affiliations:** FibEnTech Research Unit, Faculty of Engineering, University of Beira Interior, 6200-001 Covilhã, Portugal

**Keywords:** colorimetric indicator, biobased, bacterial pigments, violacein, prodigiosin, flexirubin-type pigment, bacterial cellulose, pH indicator, halochromic material, real-time quality monitoring

## Abstract

Nowadays, worldwide challenges such as global warming, pollution, unsustainable consumption patterns, and scarcity of natural resources are key drivers toward future-oriented bioeconomy strategies, which rely on renewable biobased resources, such as bacterial pigments and bacterial cellulose (BC), for materials production. Therefore, the purpose of this study was to functionalize bacterial cellulose with violacein, flexirubin-type pigment, and prodigiosin and test their suitability as pH indicators, due to the pigments’ sensitivity to pH alterations. The screening of the most suitable conditions to obtain the BC-pigment indicators was achieved using a full factorial design, for a more sustainable functionalization process. Then, the pH response of functionalized BC to buffer solutions was assessed, with color changes at acidic pH (BC-violacein indicator) and at alkaline pH (BC-violacein, BC-prodigiosin, and BC-flexirubin-type pigment indicators). Moreover, the indicators also revealed sensitivity to acid and base vapors. Furthermore, leaching evaluation of the produced indicators showed higher suitability for aqueous foods. Additionally, color stability of the functionalized BC indicators was carried out, after light exposure and storage at 4 °C, to evaluate the indicators’ capacity to maintain color/sensitivity. Thus, BC membranes functionalized with bacterial pigments have the potential to be further developed and used as pH indicators.

## 1. Introduction

Packaging technology evolves around consumers’ demands for freshness, safety, and high quality of food products, which leads to the development of new packaging solutions [1,2]. Active food packaging materials contain bioactive compounds, such as antimicrobials [3] and antioxidants [4], to extend shelf life, maintain the quality, and stability of food-packaged goods [5]. On the other hand, intelligent food packaging solutions are intended to monitor food conditions, or the environment surrounding it, detect physical, chemical, and/or biological changes, and provide a response. The response produced is designed to yield an immediate assessment of food quality [6,7,8].

Intelligent sensor-based packaging materials can be classified, taking into account the variables controlled, into time–temperature, gas, and freshness indicators, to monitor unwanted temperature fluctuations along the supply chain, variation of the gas composition in the headspace of the food package, especially in modified atmosphere packaging, and freshness decay, through changes in the concentration of metabolites indicators of microbial growth and, therefore, altering the pH [2,9]. The most common freshness indicators are composed of a solid support and a dye, sensitive to pH variations, providing a visual response through color change, depending on the pH of the environment inside the package [2,6]. Some synthetic dyes have been explored as pH indicators in several studies [10,11,12]; nonetheless, leaching of the dye and the awareness of the harmful effects generated by chemically produced dyes raises concerns among consumers, due to their bioaccumulation and toxicity [2,6,13]. Thus, natural dyes, from different sources, appear as a promising alternative, due to their biodegradability, non-toxicity, non-carcinogenicity, and their environmentally friendly production [14,15]. Examples of natural pigments in colorimetric indicator systems include anthocyanins from saffron (*Crocus sativus* L.), barberry (*Berberis vulgaris* L.), black carrots (*Daucus carota* L.), and red cabbage (*Brassica oleraceae*) [7,8,16,17], red naphthoquinone pigment shikonin from the root of gromwell (*Lithospermum erythrorhizon*) [18], curcumin from the rhizomes of turmeric (*Curcuma longa Linn.*) [19], among others. Concerning the production source, the aforementioned examples of natural pigments are from vegetable/plant origin; nonetheless, natural pigments derived from microorganisms present some advantageous features such as the shorter life cycle, which is translated into shorter productions times, no seasonal restrictions, and ease for genetic modification [20,21]. Among them, pigments of bacterial origin, such as prodigiosin, violacein, and flexirubin-type pigment are sensitive to pH alterations and therefore are promising compounds to be explored for pH indicators and food monitoring applications. 

Moreover, regarding the solid support for the development of calorimetric sensors, the environmental concerns associated with the use of non-renewable synthetic materials, which leads to considerable amounts of post-consumer waste, urges packaging solutions and the packaging industry towards sustainable, renewable and biodegradable materials [8]. Bacterial cellulose (BC) is among the polysaccharides and proteins most widespread studied in the packaging field [22]. Furthermore, even though BC and plant cellulose exhibit a similar chemical structure, their morphology is distinct since BC exhibits greater flexibility and mechanical resistance, as well as its porous structure, and can be recovered with simple downstream and upstream processes, with higher purity, whereas plant cellulose recovery involves extremely polluting reactions to remove undesirable compounds (such as lignin, pectin, and hemicellulose). Therefore, BC properties make it a versatile and ecologically sustainable exopolysaccharide for application in several fields where plant cellulose could hardly be applied [23]. Additionally, BC is approved as “generally recognized as safe” (GRAS) by regulatory agencies and, in addition to a dietary fiber, has been explored in food related applications as stabilizer, thickener, and gelling agent, as well as for food packaging purposes [22,24,25,26]. 

To the best of our knowledge, despite all advantages aforementioned, no study has yet been conducted on the suitability of bacterial pigments as pH indicators for food packaging applications. Thus, the aim of the present research is to investigate the functionalization of BC membranes with the bacterial pigments prodigiosin, violacein, and flexirubin-type pigment, improve the functionalization process using full factorial design methodology, and evaluate the suitability of the produced materials as pH indicators for food packaging solutions. Additionally, the aim of using a full factorial design methodology to improve the functionalization of BC membranes is to reduce the process’s environmental impact, by establishing the ideal functionalization conditions to maximize the color strength of the functionalized samples.

## 2. Materials and Methods

Ethanol, tris(hydroxymethyl) methylamine, and dipotassium hydrogen phosphate were provided by Fisher Chemical. Sodium hydroxide (NaOH), potassium chloride, citric acid monohydrate, sodium citrate monobasic, sodium bicarbonate, peptone, Nutrient Broth (NB), tryptic soy broth (TSB), hydrochloric acid (HCl), glycerol, and glucose monohydrate were purchased from Sigma-Aldrich. Agar-agar was acquired from Labkem (Labbox, Spain) and acetone was provided by Labchem (Laborspirit, Portugal). Alfa Aesar provided the potassium dihydrogen phosphate. Kombucha Original Bio was obtained from Freshness Diagonal, Lda (Montijo, Portugal). 

### 2.1. Bacterial Resources: Production and Recovery

#### 2.1.1. Bacterial Pigments

The bacteria *Serratia plymuthica,* gently provided by Peter Askew (Industrial Microbiological Services Ltd.), was used for prodigiosin pigment production. Growth and pigment production was accomplished in peptone glycerol phosphate (PGP) medium (5 g/L peptone, 10 mL/L glycerol, 2 g/L K_2_HPO_4_, and 15 g/L agar, for solid growth), at 20 °C, in absence of light [27]. A previously reported method, using acidified ethanol, was employed for pigment extraction [28]. The bacteria *Chromobacterium violaceum,* purchased from CECT (Spanish Type Culture Collection, University of Valencia), was used for violacein pigment production. Growth and pigment production was accomplished in TSB (17 g/L casein peptone (pancreatic), 2.5 g/L dipotassium hydrogen phosphate, 2.5 g/L glucose, 5 g/L sodium chloride, 3 g/L soya peptone (papain digest), and 15 g/L agar, for solid growth), 30 °C [29]. Pigment recovery was performed, as previously described, with ethanol [30,31]. The bacteria *Chryseobacterium shigense,* purchased from DSMZ (Leibniz Institute DSMZ-German Collection of Microorganisms and Cell Cultures), was used for flexirubin-type pigment production. Growth and pigment production was accomplished in nutrient medium (5 g/L meat peptone, 3 g/L meat extract, and 15 g/L agar, for solid growth), at 30 °C [32]. The pigment extraction was accomplished with acetone, as previously reported [32].

#### 2.1.2. Bacterial Cellulose

BC production was carried out in static conditions and the fermentation medium was a combination of 8.25 g/L of commercial green tea, 8.25 g/L of commercial black tea, 70 g/L of glucose, and 10% (*v*/*v*) of Kombucha Original Bio commercial beverage (Freshness Diagonal, Lda, Montijo, Portugal). The kombucha beverage was used as pre-inoculum, due to the presence of a microbial consortia comprising of unidentified bacterial and yeast species [33]. After 7 days of fermentation, at 30 °C, the BC membranes formed at the air–liquid interface were recovered and washed, for impurities and cellular debris removal, with 0.1 M NaOH, at 80 °C, for 30 min [34,35]. Then, distilled water was used to rinse the BC membranes, to decrease the pH from the alkali washing procedure and ultimately, the membranes were dried at room temperature, until constant weight was achieved. BC structural changes that may occur due to this alkaline treatment were assessed in a previous work [36].

### 2.2. BC Functionalization with Prodigiosin, Violacein and Flexirubin-Type Pigment

BC membranes were divided in 4 cm × 4 cm squares (≈40 g/m^2^) and the functionalization baths were prepared, individually, with prodigiosin, violacein, and flexirubin-type pigment. The pigment solution with 30% over the weight of the fiber (owf) was obtained from the dried pigment powder, whereas 17.5% and 5% owf solutions were prepared from the most concentrated pigment solutions, corresponding to concentrations of 10, 5.83, and 1.67 mg/mL, respectively. The functionalization procedures were performed on the Datacolor AHIBA IR equipment (Datacolor company, USA), with each combination of functionalization temperature (40, 65, 90 °C) and functionalization duration (20, 40, and 60 min). Raise velocity, revolutions per minute, and liquor ratio were kept constant throughout the entire study, at 2 °C/min, 20 rpm, and 1:30, respectively.

### 2.3. Factorial Experimental Design and Optimization of the Variables

A 2^3^ full factorial design was used to determine the optimized conditions for BC functionalization with each pigment, individually, to obtain a higher color strength (*K*/*S*). In addition to the three independent variables at two levels, replication with central points was also included. The high and low levels defined for the independent variables, functionalization temperature (A), duration of functionalization (B), and pigment concentration (C), are given in Table 1. For the independent variable pigment concentration, the high and low levels were defined accordingly with previously reported results and preliminary tests [36]. The experimental design was generated by Design-Expert version 7.0.0 (Stat-Ease, Minneapolis, MN, USA) and the main effects between factors were determined. Data analysis was performed using the results obtained as response (color strength (*K*/*S*)) from the reflectance values determined on functionalized BC at 585 nm, 535 nm, and 450 nm, for violacein, prodigiosin and flexirubin-type pigment, respectively. Data were treated by analysis of variance (ANOVA) and multiple regression analyses, as well as graphical optimizations, for the development of mathematical models that represented the individual and interaction effects of the independent variables on the color strength. The prediction of the models was performed with 95% significance.

### 2.4. Color Evaluation

To determine the color strength (*K*/*S* values) of BC samples functionalized with prodigiosin, violacein, and flexirubin-type pigment, the samples reflectance was measured by a Datacolor 110 spectrophotometer (Datacolor company) under illuminant D65, by a 10° standard observer. *K*/*S* values were determined using the Kubelka–Munk Equation (1):

(1) $$K/S=\frac{{\left(1-R\right)}^{2}}{2R}$$

where *K* is the absorption coefficient, *S* is the scattering coefficient, and *R* represents the observed reflectance of the functionalized sample. The results were the average of at least five measurements at different positions.

The CIELAB coordinate system was also used to characterize the functionalized BC samples. Briefly, L* indicates lightness from 0 (black) to 100 (white), a* indicates changes in redness–greenness, and b* value represents yellowness–blueness. The C* value corresponds to chroma, and was calculated from Equation (2):

(2) $$C^{*}=\sqrt{{\left(a^{*}\right)}^{2}+{\left(b^{*}\right)}^{2}}$$

For the polar coordinates, hue angle value (*H*°) denotes 0 for redness, 90 for yellowness, 180 for greenness, and 270 for blueness [37,38], and is obtained through Equation (3):

(3) $$H^{{}^{\circ}}=\tan^{-1}\left(\frac{b^{*}}{a^{*}}\right)$$

### 2.5. Preparation of pH Indicator

BC-based pH indicators were prepared with each pigment, prodigiosin, violacein, and flexirubin-type pigment, using the optimal conditions obtained from Section 2.3, in the full factorial design. BC_violacein indicators were prepared at 77.90 °C, and 26.94% owf, prodigiosin functionalized_BC at 79.53 °C, and 26.81% owf, and BC indicators functionalized with flexirubin-type pigments were prepared at 88.37 °C, with 28.25% owf pigment solution. After functionalization, for 20 min, samples were washed in running water and dried, at room temperature, for further use. 

### 2.6. Response to pH Buffer Solutions

The pH buffer solutions, in the range of 1–14, were prepared by using different combinations and proportions of potassium chloride, HCl, NaOH, sodium citrate, citric acid, potassium dihydrogen phosphate, tris(hydroxymethyl) aminoethane, or sodium bicarbonate solutions. Pigment solutions, in the optimized concentrations (see Section 2.5) were mixed with the prepared pH buffer solutions (1:20) to assess color changes. Moreover, BC-pigment indicators (sample size 4 cm × 4 cm) were immersed in each buffer solution, at room temperature, and the color parameters L*, a*, and b* were evaluated using Datacolor 110 spectrophotometer (Datacolor company, USA). The total color difference (Δ*E*) was calculated as follows, by Equation (4):

(4) $$\Delta E=\sqrt{{\left(\Delta L^{*}\right)}^{2}+{\left(\Delta a^{*}\right)}^{2}+{\left(\Delta b^{*}\right)}^{2}}$$

where ΔL*, Δa*, and Δb* were the differences between color parameters of control sample and the color parameters obtained at each pH value. 

### 2.7. Sensitivity of pH Indicators to Acid and Base Vapors 

The sensitivity of the BC-pigment indicators to acid and base gases was evaluated as previously described [13,18]. Each indicator (4 cm × 4 cm) was placed with a distance of 1 cm over the top of acetic acid or an ammonia solution for 20 min. Then, the color parameters were evaluated, and the total color difference (Δ*E*) was calculated, as described in the previous section. 

### 2.8. Leaching Assessment

Food simulant solutions were used to evaluate the release of violacein, prodigiosin, and flexirubin-type pigment from the color indicator. The food simulant solutions were prepared with ethanol, at 95% to represent fatty food, at 50% for oil-in-water emulsion food, and 10% to represent aqueous food [18,39]. Each BC-pigment indicator (2 cm × 2 cm) was placed in 20 mL of each test solution, at room temperature, for 72 h, with gentle shaking. The pigments release rate was evaluated spectrophotometrically, at 535 nm for BC_prodigiosin, 585 nm for BC_violacein, and at 450 nm for BC_flexirubin indicators. 

### 2.9. Color Stability of pH Indicators Produced

The fabricated pH indicators were evaluated regarding their color stability with exposure to artificial daylight. Briefly, BC_prodigiosin, BC_violacein, and BC_flexirubin indicators were exposed to artificial daylight D65, in a light booth (Color-chex, Atlas), for 120 h. The samples *K*/*S* was obtained at regular time intervals and the extent of fading was calculated as % of discoloration (%*D*), given by the following Equation (5):

(5) $$\%D=\frac{K/S_{\mathrm{initial}}-K/S_{\mathrm{final}}}{K/S_{\mathrm{initial}}}\times 100$$

where *K*/*S_initial_* represents the samples color strength before exposure to artificial daylight and *K*/*S_final_* corresponds to the samples color strength after exposure. 

In addition to evaluation of samples discoloration, after the 120 h of artificial daylight exposure, the recovered samples were tested in the various buffer solutions (pH 1 to 14) and the color parameters were obtained as described in previous sections. 

The color stability of the three different indicators was also assessed, in the same manner, after storage at 4 °C, for 7 days. 

### 2.10. Statistical Analysis

Statistical analysis of the results was performed with software GraphPad Prism 6 software. All experiments were carried out at least in three replicates, unless otherwise stated, and obtained data were analyzed by analysis of variance (ANOVA). The differences between means were evaluated by Tukey’s multiple comparison test (*p* < 0.05), and data were expressed as mean ± standard deviation (SD).

## 3. Results and Discussion

### 3.1. Full Factorial Design for Optimization of BC Functionalization

BC functionalization with violacein, prodigiosin, and flexirubin-type pigment was optimized using a full factorial design, which allows the determination of the effect of several factors and their interactions on the response (color strength (*K*/*S*), in this study) with a minimum number of experiments, saving time and resources [40]. 

Table 2 summarizes the standard set of the experiments in coded form and the measured response, color strength (*K*/*S*). ANOVA was performed to statistically evaluate the significance of the factors and the results obtained for the models used to estimate the *K*/*S*, for each pigment, are reported in Table 3. The significant factors and interactions, for violacein and prodigiosin pigments, were temperature, duration of functionalization, pigment concentration, and the interaction between temperature and concentration. Regarding the flexirubin-type pigment, the significant factors were only the temperature and the pigment concentration. The models’ F-value of 574.15 for violacein, 146.26 for prodigiosin, and 369.86 for flexirubin-type pigment imply that all three models are significant and there is only 0.01% chance that such a large F-value is because of experimental noise. The developed regression models to predict the response variable (*K*/*S*) expressed in terms of actual factors are as follows, for violacein (Equation (6)), prodigiosin (Equation (7)), and for flexirubin-type pigment (Equation (8)) [41]:

(6) $$K/S=0.61180\;–\;1.38667\times 10^{-3}\times T+0.011917\times D+0.038173\times C+1.53067\times 10^{-3}\times T\times C$$

(7) $$K/S=-0.21627+4.54\times 10^{-3}\times T+5.125\times 10^{-3}\times D+0.02072\times C+6.72\times 10^{-4}\times T\times C$$

(8) ln(K/S)=−3,65556+0,032343×T+0.030099×C 

where *K*/*S* represents the response variable, T, D, and C represent the factors functionalization temperature, duration of functionalization, and pigment concentration, respectively.

The normal distribution of residuals, for each model, graphically shown in Figure S1, illustrates the goodness of the fit of the models, as reported in Table 3, lack of fit is not significant in any of them. The determination of the R^2^ coefficient allows the validation of the models’ accuracy [42,43]. The R^2^-value for the violacein model was calculated as 0.9892, and it implied that the sample variation of 98.92% is attributed to the independent variables, and only 1.08% of the total variation cannot be explained by the model. This indicated that the general ability and precision of the regression model were good. The predicted R^2^ of 0.9811 is in reasonable agreement with the adjusted R^2^ of 0.9875, with a difference of less than 0.2 [42,44]. Likewise, high accuracy of the regression model to predict the *K*/*S* for prodigiosin was observed, with an R^2^-value of 0.9590 and a predicted R^2^ of 0.9321, in reasonable agreement with the adjusted R^2^ (0.9525). The R^2^-value for the flexirubin-type pigment model was 0.9462, and additionally, the predicted R^2^ (0.9243) and adjusted R^2^ (0.9423) support the high correlation between the observed and the predicted values. Moreover, the plots of actual (measured) *K*/*S* values versus the predicted response values, for each pigment, Figure S2, show the data points evenly split by the 45° line, indicating a good fit between the experimental results and the outcomes of the three models [43]. Overall, these results suggest that the three regression models provide an excellent explanation between the independent variables and the response (*K*/*S*), for each pigment. Figures S3 and S4 show contour and 3D surface plots of *K*/*S* as a function of temperature and pigment concentration at low (Figures S3a,b and S4a,b) and high levels of duration (Figure S3c,d and S4c,d). The plots (Figure S3 for violacein, and Figure S4 for prodigiosin) show that both temperature and pigment concentration have a significant effect on the response and the maximum *K*/*S* is expected when temperature and concentration are at high levels. Moreover, setting factor duration at a high level (60 min) only slightly increases this effect. Figure S5 exhibits the contour and 3D surface plot of *K*/*S*, for flexirubin-type pigment, as a function of pigment concentration and temperature at the central level of duration (40 min). The significant effect of temperature on the response is evident, especially at high flexirubin-type pigment concentration. 

These findings are in agreement with those previously reported for the effect of temperature and pigment concentration on the coloristic force. Silva et al. reported temperature and concentration of a natural pigment extract (pigment extracted from bark of *Croton urucurana* Baill.), as well as their interaction, as the most significant factors in cotton and wool fabrics functionalization [45]. The high influence of temperature in polyester fabric functionalization with the bacterial pigment prodigiosin, along with the low influence of the duration of functionalization on the color strength, has also been previously reported [46].

Based on the results obtained, it is possible to verify that, the variable duration is the least significant (in violacein and prodigiosin functionalization) and not a significant factor (in flexirubin-type pigment functionalization), if it were reduced to its low level (20 min), for the BC functionalization with the three pigments, the color strength obtained would be comparable to the color strength obtained under best dyeing conditions. Additionally, for a more sustainable functionalization procedure, the goal was to minimize the temperature of the process and the pigment concentration, while maximizing the response. Therefore, the actual factor levels selected for BC samples functionalization were: 77.90 °C, and 26.94% for violacein pigment solution, 79.53 °C, and 26.81% for prodigiosin pigment solution, and 88.37 °C and 28.25% for flexirubin-type pigment solution, with all the functionalization processes performed during 20 min. The theoretical models obtained for BC functionalization with each pigment were validated by performing the functionalization procedure, in triplicate, employing the aforementioned selected conditions. The experimental results obtained, in Table 4, are quite close to the theoretical values for each model, with an average value of 5.02 ± 0.03, 2.18 ± 0.07, and 1.06 ± 0.05, of color yield (*K*/*S*) obtained with violacein, prodigiosin and flexirubin-type pigment, respectively. Hence, the models are validated. 

### 3.2. Colorimetric Parameters Evaluation 

The colorimetric properties of the functionalized BC samples were evaluated by considering the CIELAB color system [47], and the results are summarized on Table 4. The lightness parameter, which ranges from 0 (absolute black) to 100 (absolute white) was higher for flexirubin-functionalized BC, with a value of 81.23 ± 0.42, and the lower value was observed for violacein-functionalized BC, at 40.33 ± 0.24. A positive a* value indicates the redness of the color obtained, and as expected, the highest value was reported for prodigiosin-functionalized BC. A positive b* value indicates the yellowness of the sample and was only observed for flexirubin-functionalized samples (34.17 ± 4.00), whilst a negative value represents blueness, as reported for violacein-functionalized samples, with a b* value of −25.98 ± 0.78. Chroma value (C*) was very similar for all the samples, suggesting that the vividness of the three colors obtained was similar [48]. Overall, L*, a*, and b* CIELAB parameters indicated the blueness, redness, and yellowness of BC samples functionalized with violacein, prodigiosin, and flexirubin-type pigment, respectively, as also confirmed by the hue angle values [47]. 

### 3.3. pH Responsive Properties of BC Functionalized with the Bacterial Pigments

**Figure 1:**
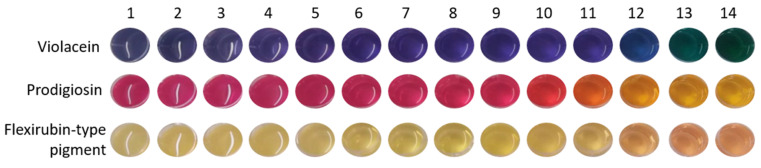
Color of violacein, prodigiosin and flexirubin-type pigment solutions at pH 1 to 14.

The color-changing properties of violacein, prodigiosin and flexirubin-type pigment solutions were tested from pH 1 to 14 and the reaction of each pigment solution under acid and base conditions are shown in Figure 1. BC functionalized with the three pigments also showed remarkable color changes, in the tristimulus color values (L*, a* and b* values), depending on the pH of buffer solutions, as shown in Table 5, Table 6 and Table 7. The total color difference (Δ*E* value) was used to evaluate the functionalized BC substantial and noticeable color changes, as required for use in pH sensing applications. 

The purple color characteristic of violacein pigment solution can be observed throughout the pH range evaluated up to buffer solution with pH 11, then a slight color change to bluish is perceived, followed by greenish colors at pH 13 and 14, Figure 1. Similar response was obtained when evaluating violacein functionalized BC with the same buffer solutions, Table 5. At pH 12, a decrease in the a* value (from 7.31 ± 0.18 to 3.78 ± 0.99) whilst maintaining a high negative b* value (−24.53 ± 0.18), indicates the blueness of the sample, and at pH 13 and 14 the a* value changes from positive (3.78 ± 0.99 at pH 12) to negative values (−14.28 ± 0.27 and −17.55 ± 2.41, at pH 13 and 14, respectively), which indicates the greenness observed. Consequently, the total color difference (Δ*E*) of these samples increased significantly. On the acidic extreme, a high color difference was also obtained, which was not perceptible from the evaluation with the pigment solution, nonetheless it was noticeable in the violacein functionalized BC with a* and b* values closer to 0, indicating a more grayish color (Table 5). 

The pink prodigiosin pigment solution showed a very perceptible color change from buffer solution at pH 9 to pH 10, Figure 1, from bright pink to a bright coral red color, followed by orange at pH 11, and yellow from 12 to 14. Regarding the prodigiosin functionalized BC, the most perceptible color change is from pH 11 to 12, with a total color difference of 22.30 ± 1.31 and 29.13 ± 1.65, respectively. Nonetheless, the increasing b* value throughout pH 6 to 14 indicates the increasing yellowness of the samples, as reflected by the increasing values in the total color difference up to 37.80 ± 0.72 at pH 14, Table 6. 

Flexirubin-type pigment solution changed color in buffer solution with pH 12, the pale yellow turned pale orange, Figure 1, and this change was also perceptible in the flexirubin functionalized BC with a decrease in b* values (from 25.77 ± 2.57 at pH 12, to 21.65 ± 0.70 at pH 13, and further reduction to 19.31 ± 0.35 at pH 14), indicating lower yellowness of the samples, Table 7, which also reflected higher color differences as given by Δ*E* values at alkaline pH (from 3.18 ± 1.28, at neutral conditions, up 18.18 ± 0.25 at pH 14). 

One of the most important parameters of color differentiation is the total color difference and previous reports indicate that standard observers do not detect color differences for *ΔE* values below 1, trained observers can detect differences between 1 and 2, but inexperienced observers can only perceive color differences between 2 and 3.5, and for values between 3.5 and 5, the color difference is evident [6,49]. Overall, the color total color difference observed in functionalized BC with the three pigments ranged from 3.18 ± 1.28 to 18.18 ± 0.25, for BC functionalized with flexirubin type pigment, from 4.21 ± 0.77 to 37.80 ± 0.72, for prodigiosin functionalized BC, and from 2.76 ± 1.29 to 40.03 ± 0.95 for violacein functionalized BC. The highest color difference values were observed in functionalized BC with prodigiosin and with violacein pigment, in alkaline conditions, and additionally, violacein functionalized BC also showed high color differences in acid conditions.

### 3.4. Sensor Response to Ammonia and Acetic Acid Vapors

Volatile amines such as trimethylamine, dimethylamine, and ammonia, generally known as total volatile basic nitrogen (TVBN), are alkaline products of enzymatic and microbial degradation during food spoilage and are widely regarded as meat spoilage indicators, as well as fish/seafood freshness indicators [9,11,19,50]. Nonetheless, in other food products, such as milk, wine, and fruit juices, food spoilage is indicated by increasing acidity [51,52]. 

Hence, the color response of the functionalized BC indicators to volatile ammonia and acid vapors was determined to validate the sensitivity to pH changes as gas sensors. The violacein functionalized BC sensor exhibited higher sensitivity towards acetic acid vapors, compared to the exposure to the ammonia vapors, Table 8, as shown by the total color difference obtained of 18.53 ± 0.54 and 5.30 ± 0.12, respectively. On the other hand, the prodigiosin-based sensor showed higher sensitivity towards ammonia vapors, with a very perceptible color change and increased yellowness value (from −5.22 ± 0.61 to 14.17 ± 0.81), as well as a higher Δ*E* value of 20.28 ± 0.61. The yellow color of the colorimetric flexirubin-type pigment-functionalized BC turned dark orange when exposed to ammonia vapor, as shown in Table 8. The decrease in b* value and a* value increase, indicate yellowness decrease and redness increase, respectively. As expected, smaller changes occurred with the exposure to acid vapors as indicated by the smaller Δ*E* of 4.05 ± 0.16, whilst the total color difference with the exposure to ammonia vapors was 13.36 ± 0.34.

Based on these results, the ammonia-sensitivity of prodigiosin and flexirubin-based sensors is an indication of their possible suitability to monitor the spoilage of meat and seafood products, while violacein-based sensors would be more fitting to monitor spoilage of milk, wine, or fruit juice products. However, further testing is required in order to evaluate the association between the number of volatile compounds produced by a certain type and quantity of food and the consequent sensor color change [18,53].

### 3.5. Release of Violacein, Prodigiosin, and Flexirubin-Type Pigment into Food Simulant Solutions

**Figure 2:**
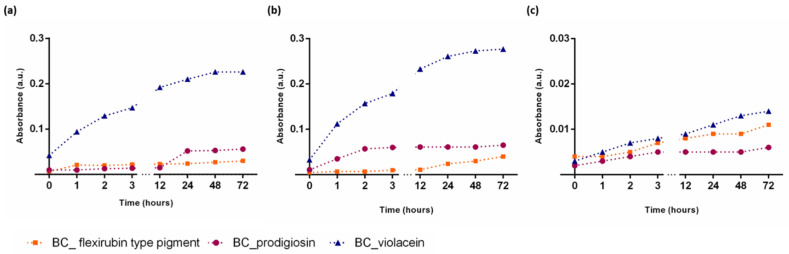
Evaluation of violacein, prodigiosin, and flexirubin-type pigment release from the color indicator in food simulant solutions: 95% ethanol, to represent fatty food (**a**); 50% ethanol, represents oil-in-water emulsion food (**b**); and 10% ethanol, to represent aqueous food (**c**). The release rate was evaluated spectrophotometrically, at 535 nm for BC_prodigiosin, 585 nm for BC_violacein, and at 450 nm for BC_flexirubin indicators.

The pigment release assessment of the functionalized BC sensors was evaluated by studying the leaching of the pigments in food simulant solutions (10, 50, and 95% ethanol), and the results are shown in Figure 2. Overall, the release of all three pigments into 50% ethanol, as an oil-in-water food simulant, occurs at a higher rate than in aqueous and fatty emulsion food simulants. Additionally, the results show that the release of violacein pigment was higher in the three food simulant solutions when compared with prodigiosin and flexirubin-type pigment, especially in the food simulant solutions with high alcohol content (50 and 95%), which can be attributed to the solubility of violacein in ethanol [54]. On the other hand, the low solubility of the bacterial pigments in water [29,32,55] also influences their release in the aqueous simulant, presenting a much lower release profile (Figure 2c), especially violacein pigment with a 16-fold decrease from the fatty food simulant and a 20-fold decrease from the oil-in-water food simulant. Therefore, the release of pigments from the BC solid support into the food simulants appears to be mainly influenced by the pigment’s solubility as well as the type of simulants, since pigments release was higher in the high alcohol content solutions. 

### 3.6. Color Stability of the pH Indicators Produced 

**Figure 3:**
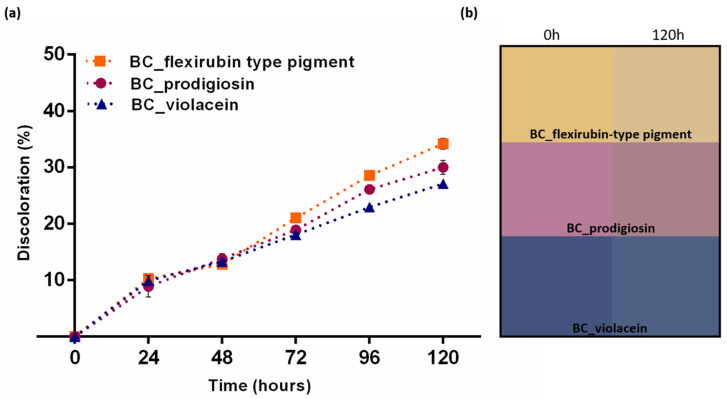
Light fastness evaluation of the pigment-functionalized BC sensors. Discoloration of each BC sensor with light exposure (**a**); apparent color shift with light exposure (**b**).

An essential criterion required in the application of pH-sensitive indicators, to provide suitable visual feedback to consumers, is the ability to sustain its color throughout the shelf-life period of the packaged goods [2]. Therefore, the impact of light and temperature exposure on functionalized BC indicators was evaluated, once natural pigments’ sensibility to external factors can lead to color loss [56] and, ultimately, impair the pH indicator performance. The pigment-functionalized BC indicators were directly exposed to artificial illumination for 120 h, uninterrupted, which contributed to the accelerated fading of the samples, as shown in Figure 3. Initially, the color fading was similar, for BC functionalized with the three different pigments; however, after 72 h of exposure, the extent of fading of each BC sample functionalized with a different pigment started to differ, which was translated into different discoloration percentages. After 120 h of exposure the higher discoloration was obtained with flexirubin-type pigment-functionalized BC, at 34.20 ± 0.96%, followed by prodigiosin functionalized BC (30.01 ± 1.24%), and the lowest discoloration was observed for BC-violacein indicator, at 27.08 ± 0.31% (Figure 3a). Figure 3b displays the apparent color of each pH indicator before and after continuous light exposure for 120 h.

As shown in Figure 3, functionalized BC indicators suffered color changes when exposed to artificial daylight for a prolonged time. Therefore, an assessment of color change in buffer solutions from pH 1-14 was performed, after samples discoloration. In addition to light exposure, the pH indicators were also stored at 4 °C, for the same amount of time, in order to assess if significant changes would occur due to temperature, and the same procedure with buffer solutions, from pH 1–14, was also performed. The results are summarized in Table 5, Table 6 and Table 7, for BC-violacein, BC-prodigiosin, and BC-flexirubin-type pigment indicators, respectively. The total color change of the control samples was obtained by comparing the functionalized BC indicators, in their natural state, before and after light exposure, as well as after storage at 4 °C. The highest color difference was observed for BC-flexirubin type pigment indicator after light exposure (12.71 ± 0.13), as expected due to the higher discoloration observed in Figure 3. BC-prodigiosin indicator exhibited a total color difference of 10.94 ± 0.32 when compared with the control sample before light exposure and the lowest color difference, after light exposure, was displayed by the BC-violacein indicator, with a *ΔE* value of 6.61 ± 0.24. Overall, the total color change observed in the control samples was higher for the samples exposed to artificial light than for the samples stored at 4 °C, with a total color difference of 6.74 ± 0.44 for BC-flexirubin type pigment indicator, 2.98 ± 0.68 for BC-prodigiosin indicator, and 5.04 ± 1.07 for BC-violacein indicator after 120 h of storage at 4 °C. 

Moreover, the total color change of the pH indicators in the different buffer solutions, after exposure to both external factors, remained similar to the behavior observed before exposure, with only a slight decrease in the total color difference, which was more noticeable for samples exposed to artificial light. Regarding BC-violacein indicators, the increased blueness was also observed at pH 12, with the decrease in the a* value, while holding a high negative b* value, as well as the greenness of samples at pH 13 and 14. Despite the color differences and an overall decrease in ΔE, due to discoloration, the BC-prodigiosin pH indicator sensitivity remained similar, with the most perceptible color change from pH 11 to 12. For the BC-flexirubin-type indicator, the decrease in yellowness at high alkaline pH was also observed, even though the color difference may not be evident due to Δ*E* values reduction, which may make the color distinction difficult [6,49]. 

## 4. Conclusions

In this study, functionalization temperature, duration, and bacterial pigment concentration were the experimental conditions investigated in order to improve the sustainability of the BC functionalization process with the bacterial pigments violacein, prodigiosin, and flexirubin-type pigment. A two-level full factorial design was used to develop mathematical models for the color strength of BC functionalized with each pigment. The R^2^ values of 0.9892 (BC-violacein), 0.9590 (BC-prodigiosin), and 0.9462 (BC-flexirubin-type pigment) indicated a good fit for the models with experimental data. The optimized functionalization process of BC with the evaluated pigments allowed the reduction of factor duration to its low level, 20 min for each process, and 77.90 °C, and 26.94% of pigment solution for BC functionalized with violacein, 79.53 °C, and 26.81% for prodigiosin, and 88.37 °C, with 28.25% pigment solution for flexirubin-type pigment. 

The functionalized BC indicators with the three distinct pigments exhibited noticeable dissimilar performances in the different assessments performed, which indicates the pH indicators’ suitability for different applications. For instance, the BC-violacein indicator showed sensitivity to acidic conditions, as well as to acetic acid vapor, with distinct color change and high Δ*E* value (18.53 ± 0.54), nonetheless, the poor performance in the leaching assessment test, especially with high alcohol content food simulants, suggests an application for aqueous foods, which could be, for example, as fruit juice spoilage indicator. On the other hand, the high sensitivity of BC-prodigiosin and BC-flexirubin-type pigment in alkaline pH and to ammonia vapors, also with a high color difference (20.28 ± 0.61, for BC-prodigiosin, and 13.36 ± 0.34 for BC_flexirubin type pigment indicator), as well as the superior performance in the leaching assessment test with the three food simulant solutions evaluated, showed the indicators’ suitability to monitor meat or fish/seafood spoilage trough TVBN. Still, further assessment is required for each approach suggested.

Moreover, the developed pH indicators could be used as stand-alone indicators, for specific functions, or an array could be fabricated, also with the incorporation of other pigments showing pH sensibility at a neutral pH level, in order to facilitate consumers’ assessment of packaged foods and a broader range of applications. Thus, bacterial pigments should be further explored for intelligent food packaging applications, due to their pH sensitivity and their inherent advantageous properties such as non-toxicity and biodegradability.

## Figures and Tables

**Table 1:** Factors and levels used in the two-level full factorial design. Additionally, three replications were performed at the central point.

Factors	Units	Code	Low Level (−1)	High Level (+1)
Functionalization temperature	°C	A	40	90
Functionalization duration	min	B	20	60
Pigment concentration	%	C	5	30

**Table 2:** Composition of the runs of the two-level full factorial design for each pigment and experimental responses (*K*/*S*).

Pigment	Run	Independent Variables			Response: Color Strength (*K*/*S*)				
					Replicates			Mean	SD
		A	B	C	I	II	III		
Violacein	1	−1	−1	−1	1.55	1.22	1.55	1.44	0.190526
	2	−1	−1	1	3.61	3.59	4.05	3.75	0.260000
	3	−1	1	−1	1.63	1.61	1.62	1.62	0.010000
	4	−1	1	1	4.35	4.07	4.42	4.28	0.185203
	5	1	−1	−1	1.36	1.49	1.71	1.52	0.176918
	6	1	−1	1	6.40	5.79	5.71	5.97	0.377403
	7	1	1	−1	2.15	2.05	2.30	2.17	0.125831
	8	1	1	1	6.47	6.46	6.62	6.52	0.089629
	9	0	0	0	3.35	3.41	3.53	3.51	0.210898
	10	0	0	0	3.36	3.34	3.96		
	11	0	0	0	3.33	3.67	3.60		
Prodigiosin	1	−1	−1	−1	0.30	0.29	0.48	0.36	0.106927
	2	−1	−1	1	1.15	1.69	1.34	1.39	0.273922
	3	−1	1	−1	0.45	0.49	0.44	0.46	0.026458
	4	−1	1	1	1.83	1.62	1.96	1.80	0.171561
	5	1	−1	−1	0.70	0.78	0.86	0.78	0.080000
	6	1	−1	1	2.74	2.61	2.76	2.70	0.081445
	7	1	1	−1	0.67	0.94	0.87	0.83	0.140119
	8	1	1	1	2.94	2.98	2.97	2.96	0.020817
	9	0	0	0	1.97	1.61	1.45	1.55	0.243641
	10	0	0	0	1.78	1.64	1.48		
	11	0	0	0	1.57	1.24	1.20		
Flexirubin-type pigment	1	−1	−1	−1	0.10	0.11	0.11	0.11	0.005774
	2	−1	−1	1	0.24	0.21	0.20	0.22	0.020817
	3	−1	1	−1	0.13	0.12	0.13	0.13	0.005774
	4	−1	1	1	0.23	0.24	0.20	0.22	0.020817
	5	1	−1	−1	0.56	0.56	0.47	0.53	0.051962
	6	1	−1	1	1.50	1.06	1.43	1.33	0.236432
	7	1	1	−1	0.51	0.45	0.60	0.52	0.075498
	8	1	1	1	1.40	1.13	1.02	1.18	0.195533
	9	0	0	0	0.28	0.25	0.27	0.36	0.072648
	10	0	0	0	0.40	0.38	0.35		
	11	0	0	0	0.44	0.39	0.44		

Note: the mean and SD values for the center point include the nine results obtained from the three runs 9, 10 and 11, for each pigment.

**Table 3:** Summary of ANOVA for the models based on full factorial design.

Pigment	Source	Sum of Squares	Degree of Freedom	Mean Square	F-Value	*p*-Value	Remarks
Violacein	Model	87.60	4	21.90	574.15	<0.0001	Significant
	Temperature	9.68	1	9.68	253.71	<0.0001	Significant
	Duration	1.36	1	1.36	35.74	<0.0001	Significant
	Concentration	71.07	1	71.07	1863.20	<0.0001	Significant
	Temperature/Concentration	5.49	1	5.49	143.96	<0.0001	Significant
	Curvature	0.063	1	0.063	1.65	0.2107	Not significant
	Residual	0.95	25	0.038	-	-	-
	Lack of Fit	0.79	19	0.041	1.48	0.3281	Not significant
	Pure Error	0.17	6	0.028	-	-	-
Prodigiosin	Model	20.85	4	5.21	146.26	<0.0001	Significant
	Temperature	3.99	1	3.99	111.84	<0.0001	Significant
	Duration	0.25	1	0.25	7.08	0.0134	Significant
	Concentration	15.55	1	15.55	436.43	<0.0001	Significant
	Temperature/Concentration	1.06	1	1.06	29.70	<0.0001	Significant
	Curvature	0.12	1	0.12	3.50	0.0731	Not significant
	Residual	0.89	25	0.036	-	-	-
	Lack of Fit	0.66	19	0.035	0.93	0.5913	Not significant
	Pure Error	0.23	6	0.038	-	-	-
Flexirubin-type pigment	Model	19.09	2	9.54	369.86	<0.0001	Significant
	Temperature	15.69	1	15.69	608.07	<0.0001	Significant
	Concentration	3.40	1	3.40	131.65	<0.0001	Significant
	Curvature	0.005	1	0.005	0.19	0.6654	Not significant
	Residual	0.70	27	0.026	-	-	-
	Lack of Fit	0.34	21	0.016	0.27	0.9884	Not significant
	Pure Error	0.36	6	0.060	-	-	-

**Table 4:** Apparent color, reflectance, color strength (*K*/*S*), and colorimetric parameters of BC functionalized with violacein, prodigiosin, and flexirubin-type pigment, as well as the *K*/*S* values, predicted with each regression model and the confidence intervals with 95% of significance.

		BC_Violacein	BC_Prodigiosin	BC_Flexirubin-Type Pigment
Apparent color		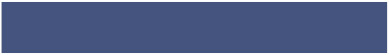	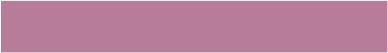	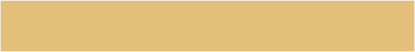
Reflectance (%R)		8.37 ± 0.05	16.12 ± 0.40	25.88 ± 0.70
Color strength (*K*/*S*)		5.02 ± 0.03	2.18 ± 0.07	1.06 ± 0.05
Color strength (*K*/*S*) predicted		4.98 95% CI [4.85, 5.12]	2.24 95% CI [2.10, 2.37]	1.05 95% CI [0.95, 1.17]
CIELAB system colorimetric parameters	L*	40.33 ± 0.24	62.73 ± 1.00	81.23 ± 0.42
	a*	6.13 ± 0.26	29.30 ± 1.28	5.83 ± 0.98
	b*	−25.98 ± 0.78	−5.22 ± 0.61	36.71 ± 3.31
	Chroma (C*)	26.69 ± 0.82	29.75 ± 1.37	34.17 ± 4.00
	Hue angle (°)	276.6 ± 11.67	349.92 ± 0.78	81.02 ± 0.74

**Table 5:** Apparent color, colorimetric parameters, and total color difference (Δ*E*) of the BC-violacein indicator at different pH values (1 to 14), first row contains initial values, second row contains the data obtained after light exposure (120 h), and the third row consist of data after storage at 4 °C (120 h).

	pH	1	2	3	4	5	6	7	8	9	10	11	12	13	14
	Control														
Initial	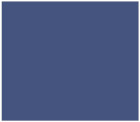	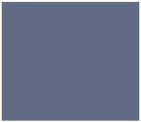	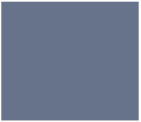	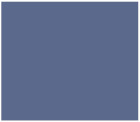	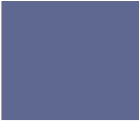	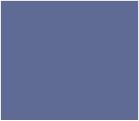	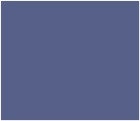	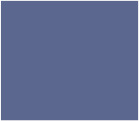	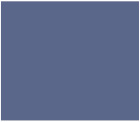	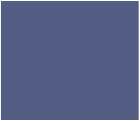	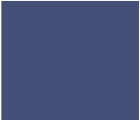	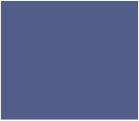	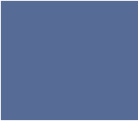	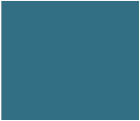	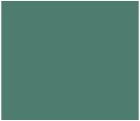
L*	40.33 ± 0.24	49.99 ± 1.34	47.83 ± 0.88	47.86 ± 0.28	48.21 ± 0.06	48.25 ± 1.18	46.76 ± 2.10	46.91 ± 1.30	45.88 ± 1.91	45.47 ± 1.94	39.50 ± 1.45	44.21 ± 0.21	48.08 ± 0.82	46.61 ± 0.59	54.11 ± 3.13
a*	6.13 ± 0.26	2.96 ± 0.22	1.48 ± 0.06	3.48 ± 0.11	6.02 ± 0.13	6.75 ± 0.02	6.81 ± 0.70	6.41 ± 0.40	5.53 ± 0.21	6.28 ± 0.17	7.03 ± 0.43	7.31 ± 0.18	3.78 ± 0.99	−14.28 ± 0.27	−17.55 ± 2.41
b*	−25.98 ± 0.78	−14.02 ± 1.33	−14.65 ± 0.97	−20.33 ± 0.20	−22.11 ± 0.09	−23.44 ± 0.14	−22.27 ± 0.37	−22.85 ± 0.86	−21.32 ± 0.60	−21.24 ± 0.13	−23.73 ± 1.22	−24.76 ± 0.40	−24.53 ± 0.18	−17.55 ± 0.09	3.06 ± 1.79
Δ*E*	0	15.70 ± 1.88	14.39 ± 0.32	9.78 ± 0.30	8.78 ± 0.09	8.34 ± 1.08	7.53 ± 1.67	7.30 ± 1.52	7.30 ± 1.82	7.05 ± 1.51	2.76 ± 1.29	4.24 ± 0.25	8.24 ± 1.09	22.96 ± 0.44	40.03 ± 0.95
After light exposure	Control														
	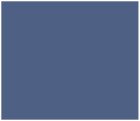	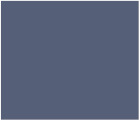	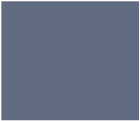	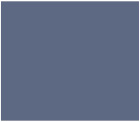	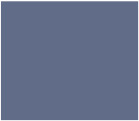	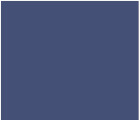	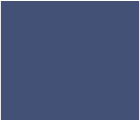	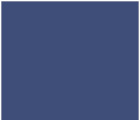	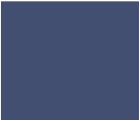	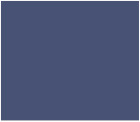	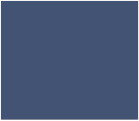	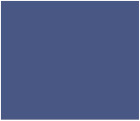	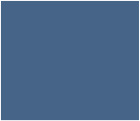	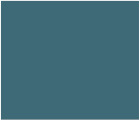	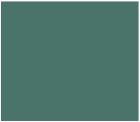
L*	44.59 ± 0.13	45.02 ± 1.29	47.18 ± 2.02	48.56 ± 1.14	49.48 ± 0.28	37.16 ± 1.95	37.92 ± 1.27	38.32 ± 0.88	38.49 ± 0.39	40.14 ± 1.05	39.84 ± 0.92	42.68 ± 1.69	42.71 ± 0.71	44.38 ± 1.82	49.00 ± 0.25
a*	3.32 ± 0.11	2.78 ± 0.25	2.54 ± 0.25	2.73 ± 0.01	3.51 ± 0.04	5.43 ± 0.26	5.65 ± 0.16	5.58 ± 0.54	4.86 ± 0.05	5.04 ± 0.06	2.68 ± 0.05	6.78 ± 0.11	0.01 ± 1.88	−11.95 ± 1.48	−16.66 ± 0.81
b*	−21.79 ± 0.42	−13.64 ± 1.06	−14.09 ± 1.20	−14.64 ± 0.41	−16.15 ± 0.37	−21.89 ± 0.21	−22.19 ± 0.03	−24.21 ± 0.79	−19.78 ± 0.45	−19.78 ± 0.45	−20.11 ± 0.40	−25. 81 ± 0.20	−22.60 ± 0.22	−11.20 ± 0.15	1.53 ± 0.76
Δ*E*	6.61 ± 0.24	8.23 ± 1.14	8.24 ± 1.78	8.22 ± 0.91	7.47 ± 0.46	7.74 ± 1.80	7.07 ± 1.25	7.09 ± 1.22	6.57 ± 0.30	5.21 ± 0.70	5.08 ± 0.99	5.75 ± 0.63	3.93 ± 1.88	18.64 ± 1.27	31.03 ± 0.09
After storage at 4 °C	Control														
	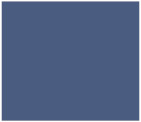	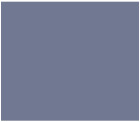	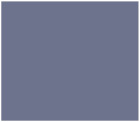	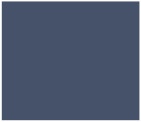	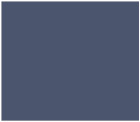	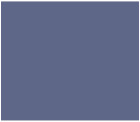	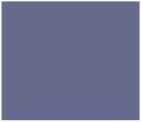	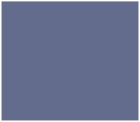	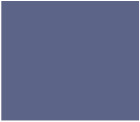	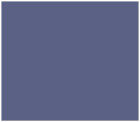	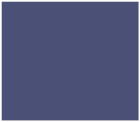	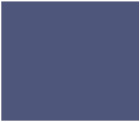	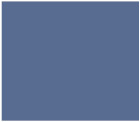	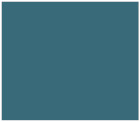	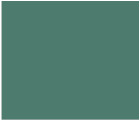
L*	42.61 ± 0.07	54.62 ± 0.82	51.35 ± 0.90	39.89 ± 1.56	39.10 ± 1.42	48.20 ± 0.86	48.61 ± 1.76	49.21 ± 0.49	47.03 ± 0.47	45.32 ± 0.46	41.19 ± 2.33	42.36 ± 1.32	48.74 ± 0.12	45.38 ± 0.25	53.39 ± 2.35
a*	3.39 ± 0.40	3.21 ± 0.16	4.89 ± 0.11	1.33 ± 0.11	3.29 ± 0.06	5.19 ± 0.33	6.34 ± 0.44	5.96 ± 0.35	5.98 ± 0.07	6.38 ± 0.10	7.25 ± 0.28	6.30 ± 0.35	0.52 ± 1.28	−13.59 ± 0.33	−18.57 ± 1.49
b*	−22.44 ± 1.17	−13.80 ± 0.52	−14.96 ± 0.82	−13.93 ± 0.76	−15.32 ± 0.76	−18.01 ± 0.82	−18.66 ± 0.29	−18.85 ± 0.02	−19.01 ± 0.78	−19.46 ± 0.37	−20.64 ± 0.18	−20.54 ± 0.41	−20.30 ± 0.91	−14.43 ± 1.63	3.35 ± 1.63
Δ*E*	5.04 ± 1.07	14.80 ± 0.97	11.60 ± 1.19	9.26 ± 0.26	8.03 ± 0.05	7.37 ± 1.06	7.73 ± 1.39	7.95 ± 0.29	6.17 ± 0.81	5.03 ± 0.41	4.75 ± 0.86	3.62 ± 0.16	7.17 ± 0.68	19.01 ± 0.43	35.61 ± 097

**Table 6:** Apparent color, colorimetric parameters, and total color difference (*ΔE*) of the BC-prodigiosin indicator at different pH values (1 to 14), first row contains initial values, second row contains the data obtained after light exposure (120 h), and the third row consist of data after storage at 4 °C (120 h).

	pH	1	2	3	4	5	6	7	8	9	10	11	12	13	14
	Control														
Initial	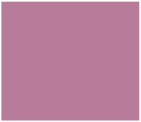	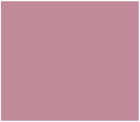	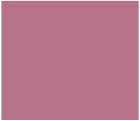	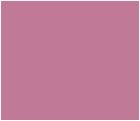	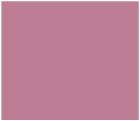	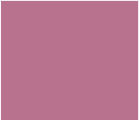	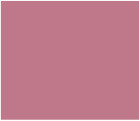	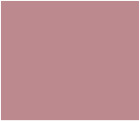	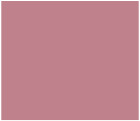	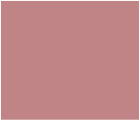	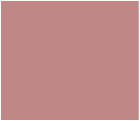	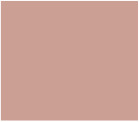	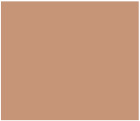	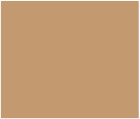	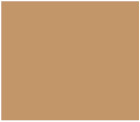
L*	62.73 ± 1.00	65.97 ± 0.54	59.93 ± 0.30	64.08 ± 1.94	62.04 ± 1.34	62.00 ± 3.06	61.98 ± 0.24	63.89 ± 1.99	64.67 ± 0.88	64.75 ± 0.99	64.90 ± 0.63	71.19 ± 1.29	68.12 ± 0.49	67.47 ± 2.26	67.69 ± 0.66
a*	29.3 ± 1.28	27.23 ± 0.87	33.20 ± 0.29	33.26 ± 1.81	31.27 ± 1.70	31.42 ± 3.80	32.06 ± 1.07	24.72 ± 2.82	25.46 ± 2.68	26.10 ± 0.43	23.07 ± 0.45	17.89 ± 0.46	19.38 ± 1.73	14.44 ± 1.15	15.17 ± 1.41
b*	−5.22 ± 0.61	−0.20 ± 0.91	−1.74 ± 0.92	−2.74 ± 1.69	−1.86 ± 0.30	−2.18 ± 0.49	2.33 ± 0.03	4.71 ± 0.78	6.07 ± 1.62	8.82 ± 1.39	9.72 ± 0.50	11.96 ± 0.76	21. 62 ± 1.06	27.03 ± 0.18	29.47 ± 0.11
*ΔE*	0	6.33 ± 1.28	5.96 ± 0.49	5.35 ± 0.07	4.21 ± 0.77	4.98 ± 1.77	8.10 ± 0.36	11.18 ± 2.06	12.17 ± 2.49	14.57 ± 1.29	16.34 ± 0.55	22. 30 ± 1.31	29.13 ± 1.65	35.86 ± 0.93	37.80 ± 0.72
After Light exposure	Control														
	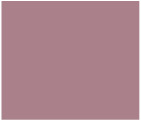	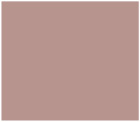	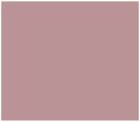	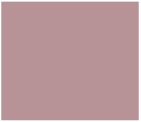	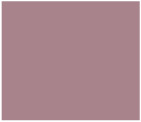	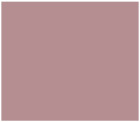	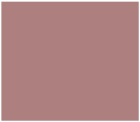	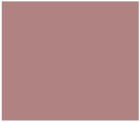	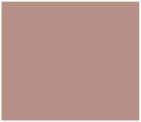	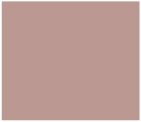	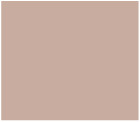	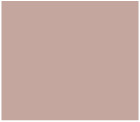	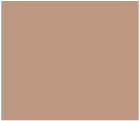	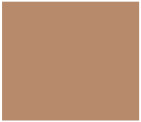	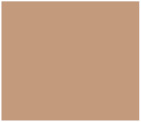
L*	61.36 ± 0.28	67.99 ± 0.92	66.79 ± 1.19	67.17 ± 0.07	62.50 ± 0.76	65.84 ± 0.18	61.37 ± 0.27	62.39 ± 0.28	66.23 ± 0.37	68.98 ± 0.20	71.61 ± 1.36	72.55 ± 0.79	68.18 ± 0.47	64.17 ± 0.01	69.12 ± 1.00
a*	20.48 ± 0.28	14.96 ± 0.24	17.25 ± 0.58	16.34 ± 0.83	17.06 ± 0.76	17.80 ± 0.66	20.27 ± 0.18	20.30 ± 0.60	15.28 ± 0.74	13.63 ± 0.35	13.33 ± 1.53	12.12 ± 0.16	13.66 ± 1.13	15.76 ± 0.23	14.11 ± 0.84
b*	1.1 ± 0.23	7.87 ± 0.69	5.06 ± 0.04	4.16 ± 0.62	2.46 ± 0.40	3.85 ± 1.41	7.87 ± 0.19	7.93 ± 0.54	9.99 ± 0.67	9.91 ± 1.15	10.10 ± 1.14	8.72 ± 0.04	16.26 ± 1.65	22.75 ± 0.88	21.84 ± 0.20
Δ*E*	10.94 ± 0.32	10.99 ± 0.25	7.48 ± 1.09	7.78 ± 0.74	3.87 ± 1.03	5.97 ± 0.81	6.77 ± 0.18	6.92± 0.59	11.39 ± 1.02	13.53 ± 0.81	15.40 ± 2.28	15.92 ± 0.62	17.97 ± 2.00	22.34 ± 0.90	23.06 ± 0.39
After storage at 4 °C	Control														
	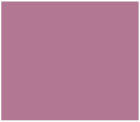	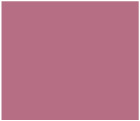	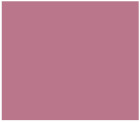	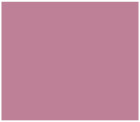	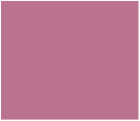	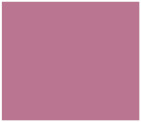	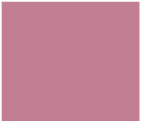	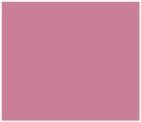	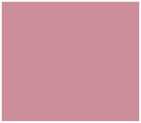	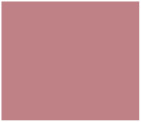	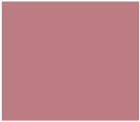	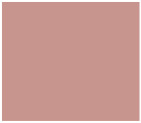	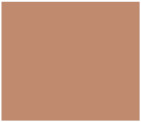	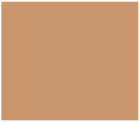	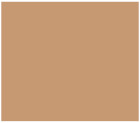
L*	60.19 ± 0.16	59.81 ± 1.71	62.48 ± 2.06	63.70 ± 0.34	60.87 ± 0.44	63.83 ± 0.37	63.72 ± 0.57	62.88 ± 2.74	67.01 ± 2.34	63.46 ± 0.10	63.05 ± 0.83	68.38 ± 1.22	66.62 ± 1.63	68.93 ± 0.61	69.11 ± 0.78
a*	30.67 ± 1.14	33.07 ± 1.71	30.64 ± 1.73	29.62 ± 0.29	33.00 ± 1.70	33.34 ± 1.22	31.14 ± 0.06	35.23 ± 1.15	29.56 ± 2.74	27.44 ± 0.30	26.73 ± 0.81	21.65 ± 0.40	20.36 ± 0.34	17.59 ± 0.64	16.12 ± 1.07
b*	−5.35 ± 0.54	0.59 ± 0.13	−0.12 ± 1.13	−1.08 ± 0.23	−2.19 ± 0.01	−2.14 ± 0.09	1.61 ± 0.34	0.72 ± 0.08	3.98 ± 0.33	7.29 ± 0.18	8.46 ± 0.16	11.47 ± 0.20	21.71 ± 1.20	27.40 ± 0.37	27.95 ± 1.48
Δ*E*	2.98 ± 0.68	6.63 ± 0.61	6.07 ± 0.19	5.63 ± 0.44	4.13 ± 0.88	4.30 ± 0.64	7.83 ± 0.05	8.28 ± 0.21	11.80 ± 1.87	13.45 ± 0.22	14.65 ± 0.53	20.78 ± 0.49	29.69 ± 0.86	36.33 ± 0.26	37.44 ± 0.72

**Table 7:** Apparent color, colorimetric parameters, and total color difference (Δ*E*) of the BC-flexirubin type pigment indicator at different pH values (1 to 14), first row contains initial values, second row contains the data obtained after light exposure (120 h), and the third row consist of data after storage at 4 °C (120 h).

	pH	1	2	3	4	5	6	7	8	9	10	11	12	13	14
	Control														
Initial	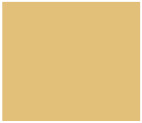	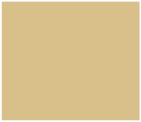	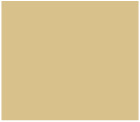	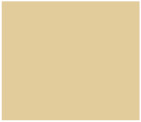	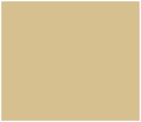	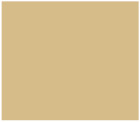	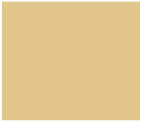	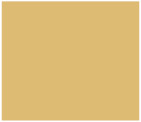	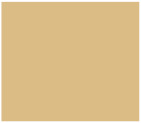	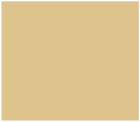	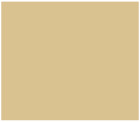	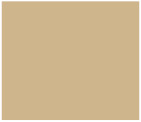	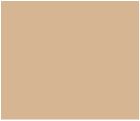	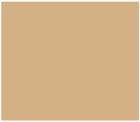	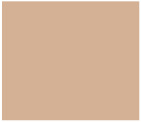
L*	81.23 ± 0.42	79.78 ± 1.06	80.83 ± 0.24	83.79 ± 1.34	80.24 ± 0.60	79.36 ± 0.23	82.96 ± 0.24	80.06 ± 0.58	81.12 ± 1.61	82.07 ± 0.13	82.13 ± 0.80	77.36 ± 0.00	79.18 ± 3.41	79.15 ±1.33	76.69 ± 0.35
a*	5.83 ± 0.98	5.15 ± 0.39	4.24 ± 0.40	4.24 ± 1.29	4.28 ± 0.91	5.37 ± 0.45	4.94 ± 0.37	6.47 ± 0.62	5.54 ± 0.84	4.93 ± 0.23	3.79 ± 0.41	6.51 ± 0.25	7.96 ± 2.84	9.06 ± 1.21	11.29 ± 0.54
b*	36.71 ± 3.31	29.91 ± 0.59	29.63 ± 0.26	29.58 ± 4.43	29.36 ±2.50	30.15 ± 1.14	33.23 ± 1.32	39.59 ± 1.04	30.45 ± 1.99	29.50 ± 1.85	27.00 ± 1.34	25.06 ± 1.12	25.77 ± 2.57	21.65 ± 0.70	19.31 ± 0.35
Δ*E*	0	7.04 ± 0.39	7.28 ± 0.33	7.75 ± 4.78	7.61 ± 2.52	6.86 ± 1.06	4.00 ± 1.34	3.18 ± 1.28	6.40 ± 1.96	7.32 ± 1.86	9.97 ± 1.46	12.30 ± 1.05	11.86 ± 1.27	15.60 ± 0.25	18.18 ± 0.25
After light exposure	Control														
	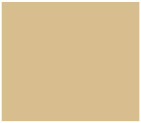	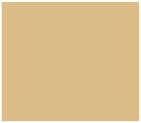	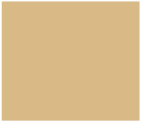	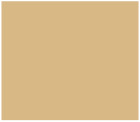	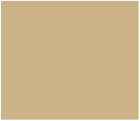	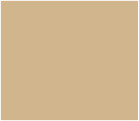	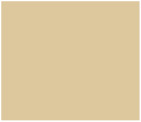	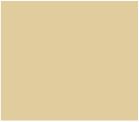	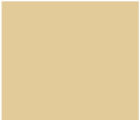	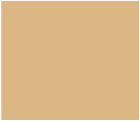	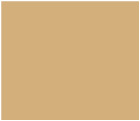	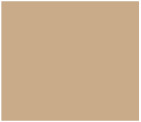	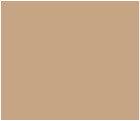	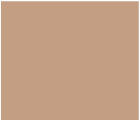	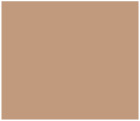
L*	81.19 ± 0.21	80.10 ± 0.53	79.68 ± 0.52	78.98 ± 0.01	76.29 ± 0.13	77.78 ± 0.36	83.75 ± 0.86	84.91 ± 0.40	84.60 ± 0.40	79.38 ± 0.38	75.79 ± 0.22	73.81 ± 0.91	72.62 ± 0.11	69.93 ± 0.95	69.15 ±0.06
a*	4.80 ± 0.13	7.02 ± 0.11	7.24 ± 0.16	7.20 ± 0.16	6.53 ± 0.13	6.54 ± 0.78	2.82 ± 0.64	2.89 ± 0.41	3.72 ± 0.17	8.17 ± 0.22	8.73 ± 0.17	9.85 ± 1.03	10.77 ± 0.27	13.53 ± 0.70	13.91 ± 0.10
b*	24.05 ± 0.12	31.10 ± 0.66	30.02 ± 0.74	29.99 ± 0.84	23.87 ± 1.45	22.89 ± 1.97	22.94 ± 1.70	25.09 ± 1.39	27.53 ± 0.47	29.80 ±2.06	30.56 ± 0.40	20.52 ± 1.27	21.38 ± 0.64	19.42 ± 0.57	20.37 ± 0.25
Δ*E*	12.71 ± 0.13	7.48 ± 0.74	6.65 ± 0.60	6.79 ± 0.69	5.30 ± 0.02	4.28 ± 0.08	3.55 ± 1.51	4.44 ± 0.18	5.02 ± 0.02	6.93 ± 1.91	9.33 ±0.23	9.63 ± 1.70	10.79 ± 0.10	14.99 ± 0.94	15.54 ± 0.07
After storage at 4 °C	Control														
	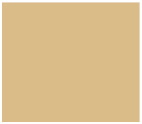	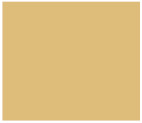	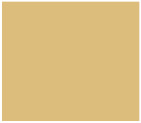	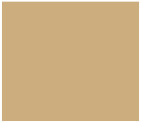	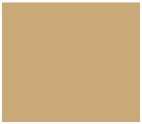	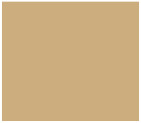	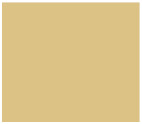	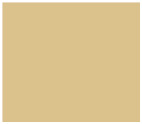	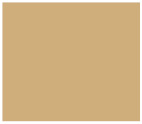	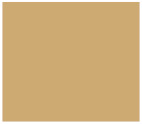	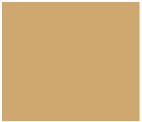	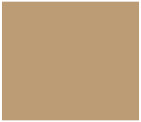	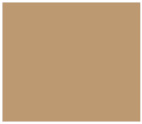	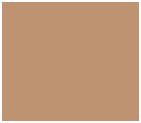	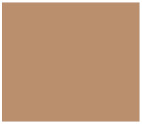
L*	79.75 ± 0.13	80.00 ± 0.37	79.95 ± 0.54	73.86 ± 1.36	74.00 ± 0.46	75.19 ± 0.43	80.90 ± 0.54	81.63 ± 0.21	74.57 ± 1.28	74.34 ± 0.23	73.92 ± 0.36	68.73 ± 0.37	67.94 ± 0.73	67.24 ± 0.16	66.68 ± 1.01
a*	6.62 ± 0.23	6.39 ± 0.09	6.77 ± 0.74	8.54 ± 0.61	8.61 ± 0.23	8.00 ± 0.18	4.33 ± 0.39	4.31 ± 0.05	9.53 ± 0.59	9.52 ± 0.27	9.83 ± 0.17	9.15 ± 0.33	11.15 ± 0.01	14.11 ± 0.45	14.29 ± 0.48
b*	30.19 ± 0.51	37.18 ± 0.55	36.22 ± 0.13	28.03 ± 0.25	28.41 ± 0.27	28.20 ± 0.31	27.43 ± 0.51	30.41 ± 0.83	32.17 ± 2.52	32.19 ± 0.61	33.31 ± 0.97	25.81 ± 1.55	24.57 ± 0.45	25.24 ± 1.57	22.36 ± 2.25
Δ*E*	6.74 ± 0.44	7.00 ± 0.56	6.07 ± 0.13	6.58 ± 1.31	6.35 ± 0.41	5.18 ± 0.31	3.77 ± 0.77	3.05 ± 0.15	6.44 ± 2.09	6.47 ± 0.50	7.37 ± 0.77	12.18 ± 0.16	13.85 ± 0.81	15.44 ± 0.59	17.16 ± 0.04

**Table 8:** Apparent color, colorimetric parameters, and total color difference (Δ*E*) of the color indicators response to ammonia and acetic acid vapors.

		Acetic Acid Vapors	Control	Ammonia Vapors
BC_violacein		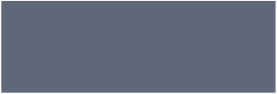	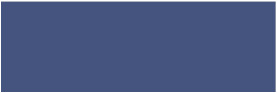	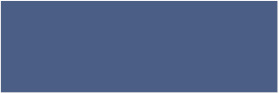
	L*	47.98 ± 0.86	40.33 ± 0.24	43.48 ± 0.28
	a*	0.88 ± 0.01	6.13 ± 0.26	2.85 ± 0.02
	b*	−9.95 ± 0.22	−25.98 ± 0.78	−23.27 ± 0.13
	Δ*E*	18.53 ± 0.54	0	5.30 ± 0.12
BC_prodigiosin		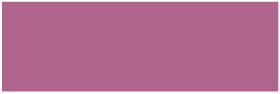	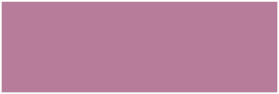	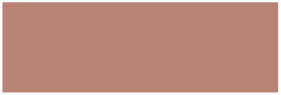
	L*	56.70 ± 1.08	62.73 ± 1.00	62.58 ± 1.25
	a*	36.68 ± 1.56	29.3 ± 1.28	23.42 ± 0.59
	b*	−6.16 ± 0.93	−5.22 ± 0.61	14.17 ± 0.81
	Δ*E*	9.59 ± 1.97	0	20.28 ± 0.61
BC_flexirubin-type pigment		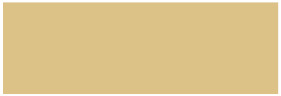	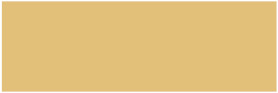	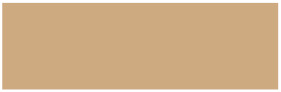
	L*	81.50 ± 0.13	81.23 ± 0.42	74.08 ± 0.40
	a*	4.70 ± 0.00	5.83 ± 0.98	10.47 ± 0.80
	b*	32.84 ± 0.16	36.71 ± 3.31	26.44 ± 0.19
	Δ*E*	4.05 ± 0.16	0	13.36 ± 0.34

## Data Availability

Data are contained within the article, Supplementary Material, and doi.org/10.18502/kms.v7i1.11608.

## Supplementary Materials

The following supporting information can be downloaded at: https://www.mdpi.com/article/10.3390/polym14183869/s1, Figure S1: Normal probability plot of internally studentized residuals, Figure S2: Plots of actual vs. predicted *K*/*S*, for each pigment, Figure S3: Contour and 3D response surface plots for interactive effects of variables on the *K*/*S* of violacein pigment, Figure S4. Contour and 3D response surface plots for interactive effects of variables on the *K*/*S* of prodigiosin pigment, Figure S5: Contour and 3D response surface plots for interactive effects of variables on the *K*/*S* of flexirubin-type pigment.
