# Supplementary material for: Design and Preparation of a Biobased Colorimetric pH Indicator from Cellulose and Pigments of Bacterial Origin, for Potential Application as Smart Food Packaging
Source: Polymers (Basel). 2022 Sep 15;14(18):3869. doi: 10.3390/polym14183869 (PMC9506293; doi:10.3390/polym14183869)
Supplement: Supplementary file 1 [file polymers-14-03869-s001.zip › polymers-1914411-SI.pdf]

Supplementary information

Design and preparation of a biobased colorimetric pH indicator with bacterial cellulose and pigments of bacterial origin

Lúcia F. A. Amorim[1], Ana P. Gomes[2], Isabel C. Gouveia*[3]

[1]FibEnTech Research Unit, Faculty of Engineering University of Beira Interior, Covilhã, Portugal
[2]FibEnTech Research Unit, Faculty of Engineering University of Beira Interior, Covilhã, Portugal
[3]FibEnTech Research Unit, Faculty of Engineering University of Beira Interior, Covilhã, Portugal

***Corresponding author**

Isabel C. Gouveia

E-mail: igouveia@ubi.pt

Tel: +351275241388

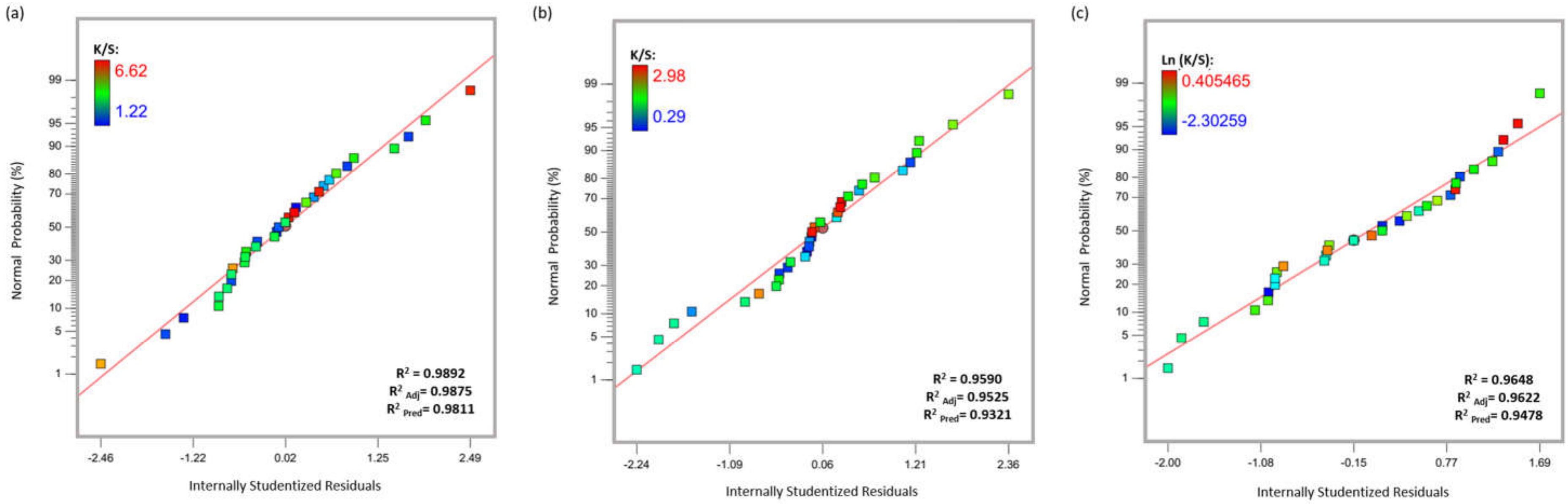

Figure S1: **Normal probability plot of internally studentized residuals** calculated from each model: violacein (a), prodigiosin (b), and Flexirubin-type pigment (c). R^2, R^2_{Adj}, and R^2_{Pred} values suggest that each model is accurately fitting the data.

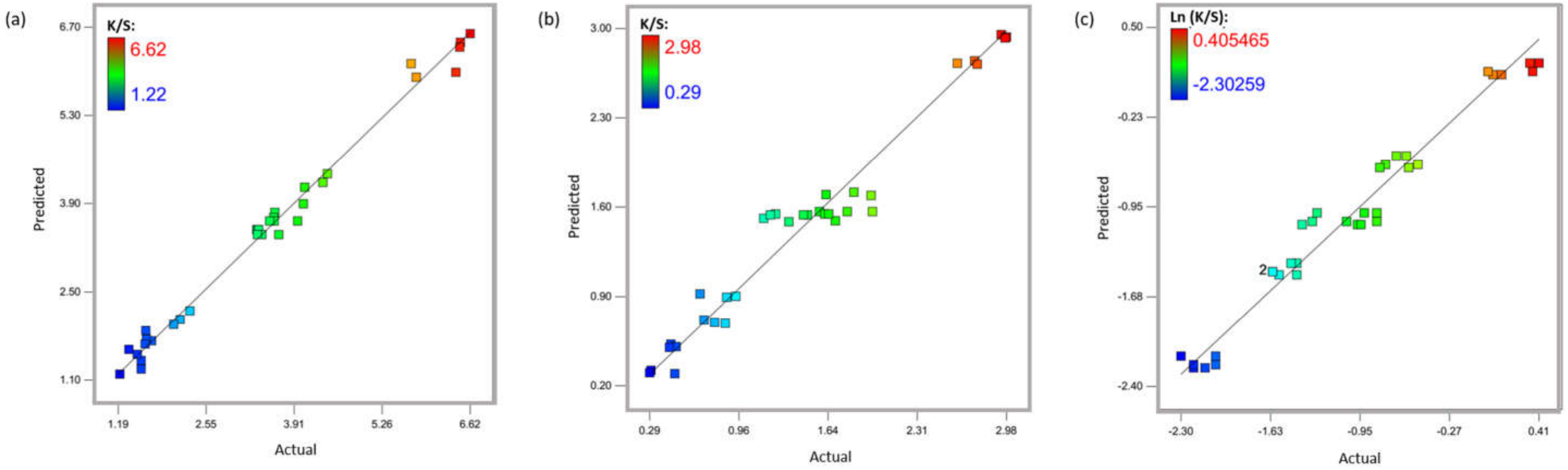

Figure S2. Plots of actual vs. predicted K/S, for each pigment: violacein (a), prodigiosin (b), and Flexirubin-type pigment (c). In each model, the data points are evenly split, suggesting a good fit between the experimental results and the model results.

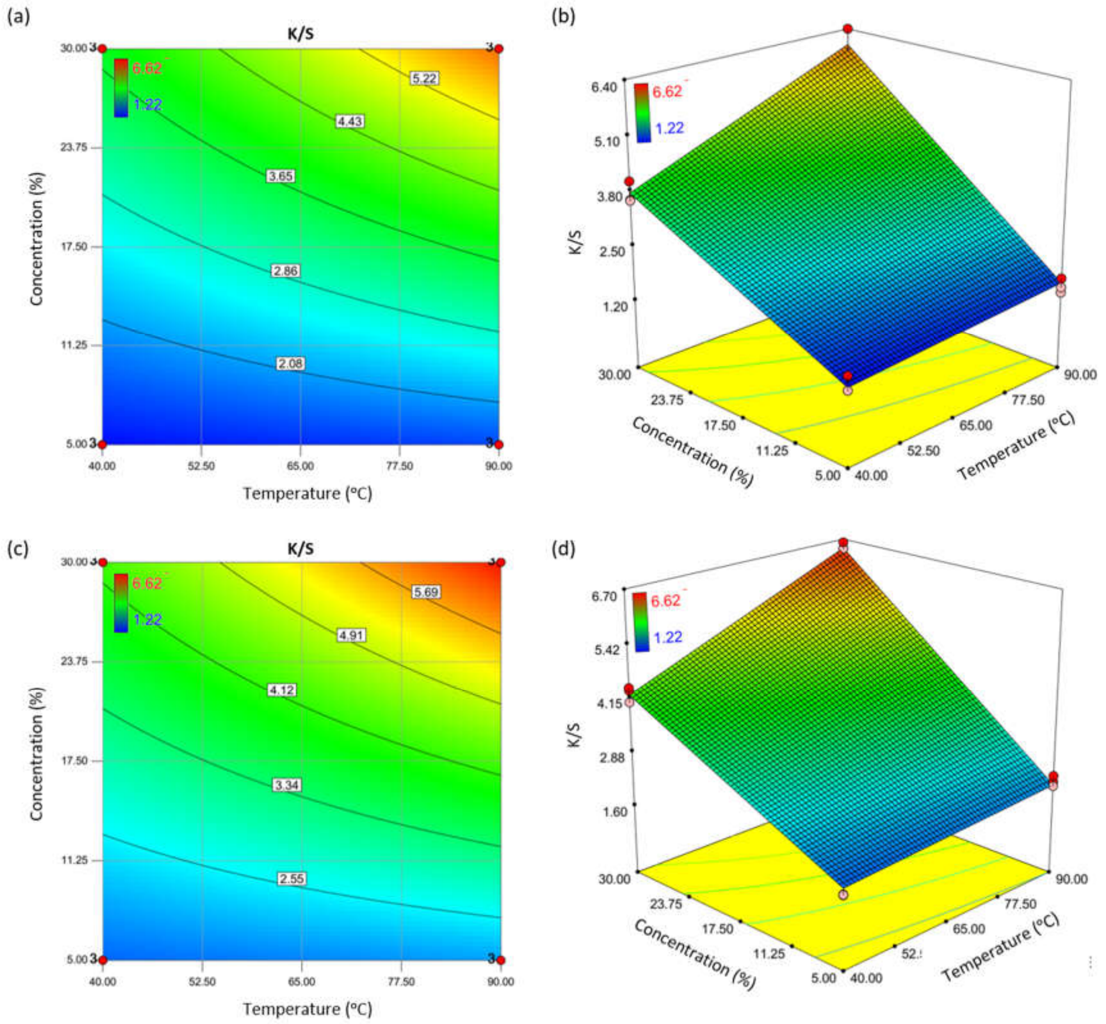

Figure S3. Contour and 3D response surface plots for interactive effects of variables on the K/S of violacein pigment at low level of factor duration (20 min) (a) and (b), and at high level of duration (60 min) (c) and (d).

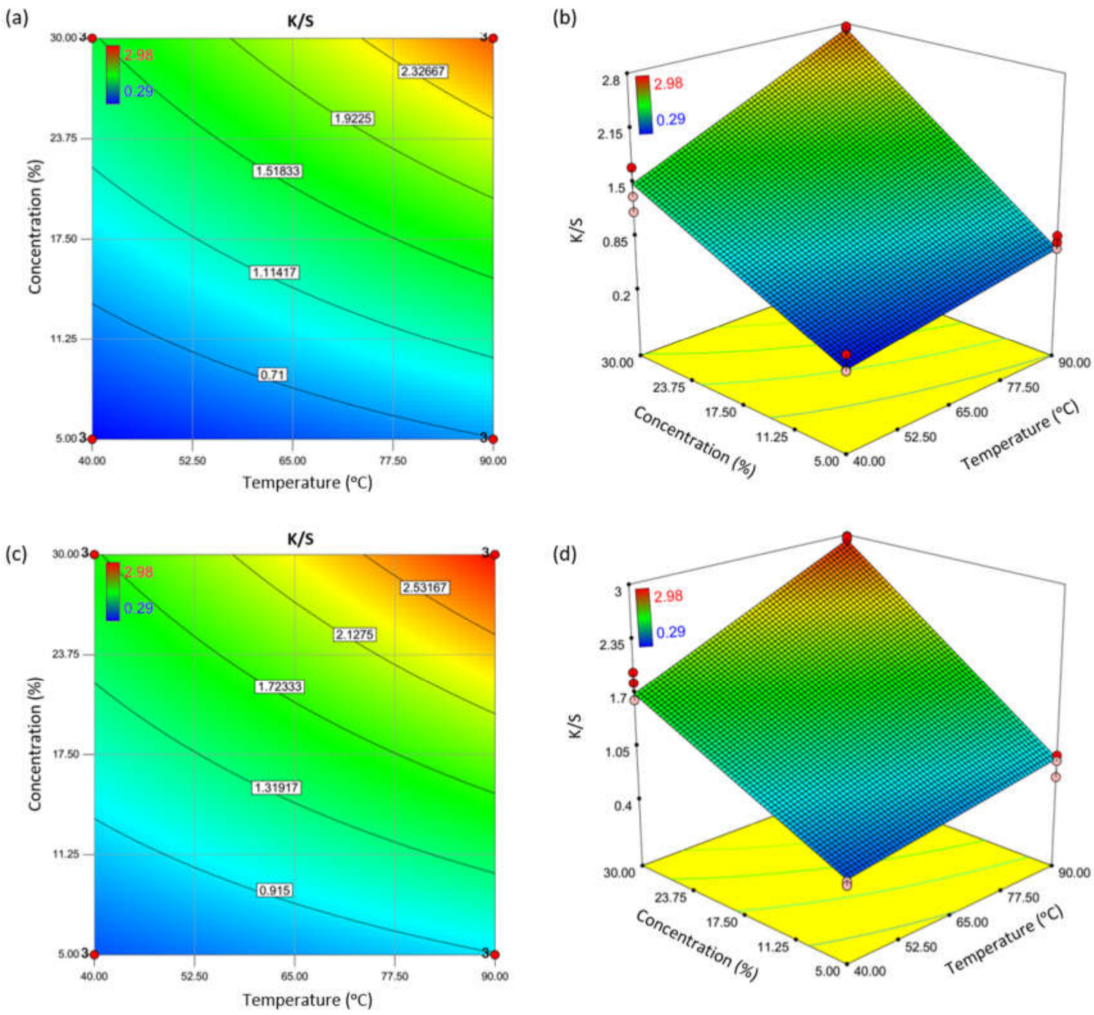

Figure S4. Contour and 3D response surface plots for interactive effects of variables on the K/S of prodigiosin pigment at low level of factor duration (20 min) (a) and (b), and at high level of duration (60 min) (c) and (d).

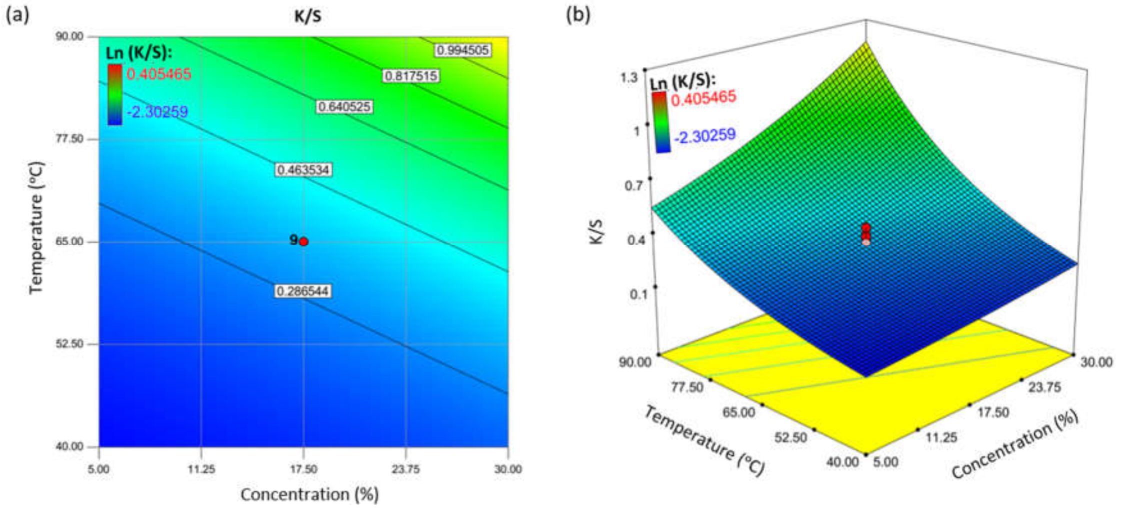

Figure S5. Contour (a) and 3D response surface (b) plots for interactive effects of variables on the K/S of flexirubin-type pigment at a fixed level of factor duration (40 min).
